# Multi-cohort gene expression model enhances prognostic stratification in diffuse large B-cell lymphoma

**DOI:** 10.1016/j.htct.2025.103847

**Published:** 2025-06-12

**Authors:** Valbert Oliveira Costa Filho, Felipe Pantoja Mesquita, Erick Figueiredo Saldanha, Pedro Robson Costa Passos, Mariana Macambira Noronha, Silvia Helena Barem Rabenhorst

**Affiliations:** a Center of Research and Drug Development (NPDM), Federal University of Ceara, Fortaleza, Brazil; b Division of Medical Oncology and Hematology, Princess Margaret Cancer Center, University Health, Biomedical Research Center, Federal University of Ceara, Fortaleza, Brazil; c Biomedical Research Center, Federal University of Ceara, Fortaleza, Brazil

Dear Editor,

Diffuse large B-cell lymphoma (DLBCL), the most common type of lymphoma, in most cases is marked by significant heterogeneity and aggressive clinical behavior. While standard chemotherapy often achieves initial responses, these are short-lived, and resistance and relapse are frequent challenges.^1^ Traditionally, risk stratification has relied on clinical tools, including the International Prognostic Index (IPI) and its variation.^2^ However, molecular stratification is promising to predict outcomes with greater accuracy, though gene-based approaches are still preliminary.^3^ Progress in this field is hindered by limited sample sizes and the substantial intra- and inter-regional variability of DLBCL.^4^^,^^5^ Consequently, large-scale studies are essential to refine risk stratification and optimize patient outcomes.

This study aimed to establish a prognostic gene expression signature for patients with DLBCL based on tumor transcriptome patterns. To achieve this, we analyzed transcriptome and survival data from 11 diverse cohorts worldwide. Given the variability in RNA sequencing or microarray platforms across the 11 datasets, we focused on the genes common to all datasets, resulting in a panel of 11,425 genes. Detailed information regarding the datasets can be found in Supplementary Table 1. Due to platform-specific differences in scale, the gene expression values were transformed into z-scores. Datasets with fewer than 100 patients were combined into a cohort referred to as the Merged Cohort. In total, six cohorts were used in this study: the National Cancer Institute Cohort (GSE10846), University of York Cohort (GSE181063), University of York II Cohort (GSE32918), Universitätsmedizin Berlin Cohort (GSE4475), University of Leeds Cohort (GSE69053), and the Merged Cohort (GSE69053, E_TABM_346, GSE11318, GSE21846, GSE23501, GSE57611, and TCGA-DLBC).

For each cohort, a univariate Cox regression was performed employing all genes in the panel, identifying those with a p-value <0.05 as prognostic. Genes were defined as core prognostic genes (CPGs) if they consistently predicted either favorable prognosis in at least 5 out of 6 cohorts or unfavorable prognosis in at least 5 out of 6 cohorts, with no conflicting outcomes.

This process led to the identification of 50 CPGs. To mitigate the risk of overfitting, a penalized Cox regression was applied using the Least Absolute Shrinkage and Selection Operator (Lasso-Cox), thereby allowing for the selection of only the most significant CPGs. The University of York cohort had the largest number of patients and was therefore used to train the Lasso-Cox model, while the other cohorts were used for validation. The final risk score was developed based on the expression levels of 22 CPGs selected through the Lasso-Cox regression (Figure 1A). The formula for calculating the risk score is as follows:

RiskScore=(β1×Gene1)+(β2×Gene2)+…+(β22×Gene22)

where ‘βX’ represents the coefficients derived from the Lasso-Cox regression, and ‘GeneX’ refers to the z-score of the expression of each gene for a given sample. The list of selected genes and their corresponding coefficients can be found in Supplementary Table 2.

**Figure 1:**
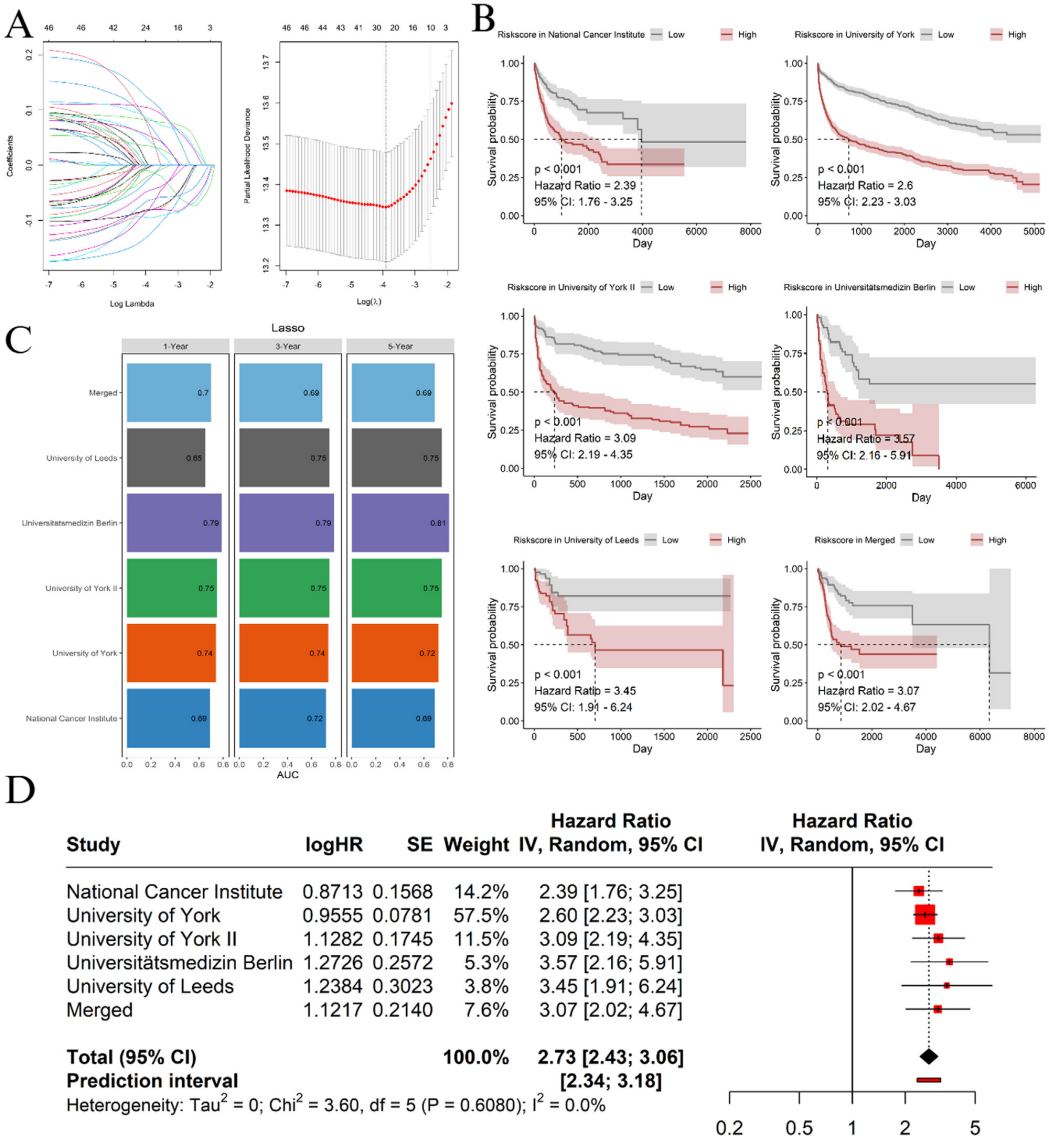
A: least absolute shrinkage and selection operator penalized cox regression feature selection. B: Kaplan-Meier analysis of the different cohorts used in this study comparing Low Risk to High Risk Groups. C: Area under the receiver operating characteristic curve (AUC) for the model in different cohort and time-point evaluations. D: Pooled analysis of the hazard ratio of being in the High Risk Group. Lasso: least absolute shrinkage and selection operator.

Patients were then divided into High Risk (> median) and Low Risk (≤ median) Groups based on the risk score. Survival analysis using Kaplan-Meier curves was conducted, revealing that the developed risk groups were significant predictors of overall survival in all cohorts (Figure 1B). Additionally, the risk score demonstrated high predictive accuracy, achieving great (≥0.69) areas under the receiver operating characteristic curve (AUC) across all cohorts (Figure 1C). By pooling the hazard ratios (HR) from the cohorts using a random effects model, the HR for death of being in the High Risk Group was 2.73 (range: 2.43–3.05; Figure 1D), further validating the risk score as a strong predictor of survival.

To ensure the prognostic value of the risk groups, even when assessed alongside clinical data, we conducted multivariable Cox regressions for each cohort. The results demonstrated that the risk groups remained strong predictors of survival. Figure 2A presents the clinical characteristics of the cohorts analyzed in this study, along with the results of the multivariate Cox regression analysis.

**Figure 2:**
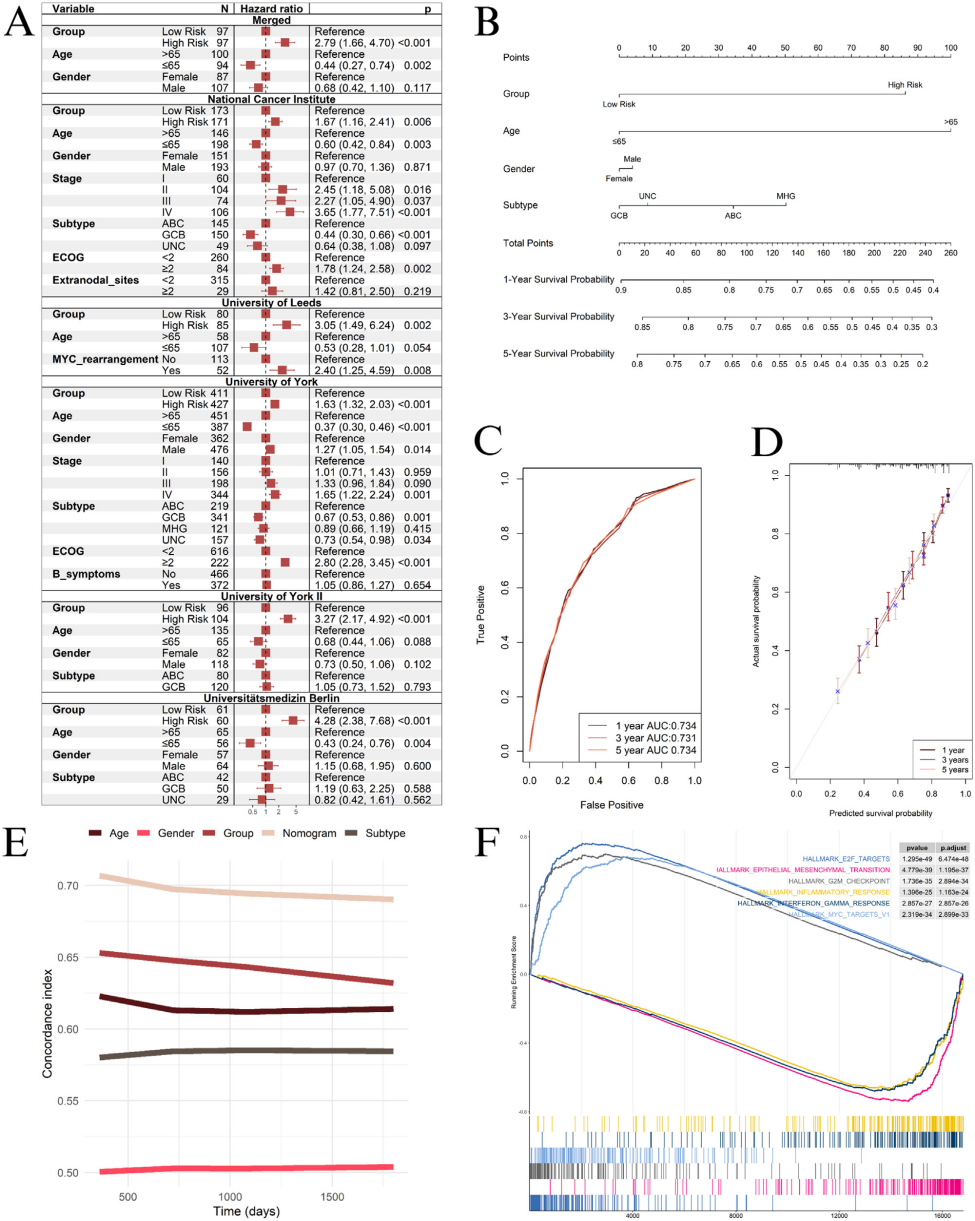
A: Multivariate cox-regression analysis of the risk groups and other available clinical information. B: Nomogram integrating our risk groups with clinical information. E: Comparison of the concordance index of our model and other variables. F: Gene set enrichment analysis plot comparing high-risk and low-risk groups in the University of York cohort. GCB: Germinal center B-cell-like; ABC: Activated B-cell-like; MHG: Molecular high-grade B-Cell lymphoma; UNC: Unclassified.

To integrate the established risk groups with other clinical variables, we developed a nomogram (Figure 2B) using a meta-cohort of patients who provided complete information on sex, age (over 65 years or 65 years and younger), and DLBCL subtype (germinal center B-cell-like, activated B-cell-like, molecular high-grade B-cell lymphoma, and unclassified), comprising a total of 2102 patients. The nomogram showed an excellent AUC for survival prediction at 1, 3, and 5 years (Figure 2C) and generated survival predictions that closely matched observed outcomes as determined by the calibration plot (Figure 2D). Moreover, the nomogram attained the highest c-index for survival prediction when compared to risk groups and clinical variables alone (Figure 2E). A free online platform has been developed and made accessible at https://costafilhoetal.shinyapps.io/CoreProgDLBCL/ to enhance the applicability of the nomogram.

We performed a Gene Set Enrichment Analysis (GSEA) using raw data from the University of York cohort and the Hallmark of Cancer gene sets from the Molecular Signatures Database (MSigDB) to better understand the biological processes distinguishing the risk groups. Notably, the GSEA (Supplementary Table 3) results revealed that the High Risk Group was predominantly enriched for E2F targets, MYC targets, and G2M checkpoint pathways, while showing downregulation of inflammatory response, interferon-gamma response, and epithelial-mesenchymal transition pathways (Figure 2F).

This study introduces a promising approach to prognostic stratification in DLBCL, utilizing gene expression data to identify CPGs and develop a validated risk score. While the IPI and its variations remain widely used for stratification in DLBCL, their discriminative power is often limited, with various studies reporting suboptimal overall survival prediction when used alone.^6^^,^^7^ Nonetheless, the European Society for Medical Oncology (ESMO) currently endorses age-adjusted IPI, which has a reported c-index of 0.613, for stratifying under 60-year-old patients who may benefit from involved-field radiotherapy or autologous stem-cell transplantation.^7^

Furthermore, neither the ESMO nor the National Comprehensive Cancer Network guidelines have incorporated transcriptomic and exome stratification in patient management.^8^^,^^9^ By outperforming traditional approaches focused on histopathology, our model was able to refine risk stratification by integrating precision oncology and shows promise in aiding treatment decisions, addressing the urgent need for improved stratification in a context where 30–50 % of DLBCL patients are not cured by standard chemotherapy.^10^ In conclusion, our global multi-cohort study represents a significant advancement in the prognostic stratification of DLBCL. The integration of this model with clinical variables enabled the development of an accurate nomogram for survival prediction. Future studies should aim to validate this model in large prospective cohorts and explore its integration into clinical practice to enhance patient outcomes.

## Conflicts of interest

The author declares no conflicts of interest

## Appendix Supplementary materials

Image, application 1

## Footnotes

Supplementary material associated with this article can be found, in the online version, at doi:10.1016/j.htct.2025.103847.
