# Supplementary material for: Multi-cohort gene expression model enhances prognostic stratification in diffuse large B-cell lymphoma
Source: Hematol Transfus Cell Ther. 2025 Jun 12;47(3):103847. doi: 10.1016/j.htct.2025.103847 (PMC12205312; doi:10.1016/j.htct.2025.103847)
Supplement: Supplementary file 1 [file mmc1.docx]

**Supplementary Table 1:** Detailed information regarding the datasets

| **Institution** | **Technology** | **Platform used** | **Accession** |
| --- | --- | --- | --- |
| National Cancer Institute | Microarray | Affymetrix Human Genome U133 Plus 2.0 Array | GSE10846 |
| University of York | Microarray | Illumina HumanHT-12 WG-DASL V4.0 R2 expression beadchip | GSE181063 |
| University of York II | Microarray | Illumina HumanRef-8 WG-DASL v3.0 | GSE32918 |
| Universitätsmedizin Berlin | Microarray | Affymetrix Human Genome U133 Plus 2.0 Array | GSE4475 |
| University of Leeds | Microarray | Illumina HumanHT-12 WG-DASL V4.0 R2 expression beadchip | GSE69053 |
| Ligue Nationale Contre le Cancer | Microarray | Affymetrix Human Genome U133A Array | E_TABM_346 |
| Lymphoid Malignancies Branch NCI | Microarray | Affymetrix Human Genome U133 Plus 2.0 Array | GSE11318 |
| Centro Nacional de Investigaciones Oncológicas | Microarray | Agilent-012391 Whole Human Genome Oligo Microarray G4112A | GSE21846 |
| University of Californa San Francisco | Microarray | Affymetrix Human Genome U133 Plus 2.0 Array | GSE23501 |
| University Hospital Essen | Microarray | Affymetrix Human Genome U133A Array | GSE57611 |
| The Cancer Genome Altas | RNA-seq | Illumina HiSeq 2000 | TCGA-DLBC |

**Supplementary Table 2:** Selected genes and their corresponding coefficients

| DFFA | 10,42308771 |
| --- | --- |
| FBP1 | -2,531140618 |
| GP2 | 5,356529356 |
| JADE3 | 8,163961972 |
| KLRC1 | -12,71868438 |
| LCP2 | -9,49491964 |
| LGALS2 | -4,607486892 |
| LST1 | -0,997687429 |
| LXN | -6,317259492 |
| MACIR | 8,448492514 |
| MMP9 | -8,002477732 |
| NCALD | -8,151977167 |
| NUPR1 | 6,774764051 |
| PIGR | 3,511298682 |

**Supplementary Table 3:** Gene Set Enrichment Analysis

| **ID** | **Enrichment Score** | **NES** | **p-value** | **p.adjust** | **q-value** |
| --- | --- | --- | --- | --- | --- |
| HALLMARK_E2F_TARGETS | 0.76 | 3.48 | 1.29E-49 | 6.47E-48 | 2.32E-43 |
| HALLMARK_EPITHELIAL_MESENCHYMAL_TRANSITION | -0.74 | 3.48 | 4.78E-39 | 1.19E-37 | 4.28E-33 |
| HALLMARK_G2M_CHECKPOINT | 0.70 | 3.20 | 1.74E-35 | 2.89E-34 | 1.04E-30 |
| HALLMARK_MYC_TARGETS_V1 | 0.69 | 3.13 | 2.32E-34 | 2.90E-33 | 1.04E-28 |
| HALLMARK_INTERFERON_GAMMA_RESPONSE | -0.68 | -2.70 | 2.86E-27 | 2.86E-26 | 1.02E-21 |
| HALLMARK_INFLAMMATORY_RESPONSE | -0.67 | -2.65 | 1.40E-25 | 1.16E-24 | 4.16E-21 |
| HALLMARK_TNFA_SIGNALING_VIA_NFKB | -0.65 | -2.57 | 2.89E-22 | 2.07E-21 | 7.40E-17 |
| HALLMARK_COMPLEMENT | -0.64 | -2.55 | 1.23E-21 | 7.67E-21 | 2.74E-16 |
| HALLMARK_ALLOGRAFT_REJECTION | -0.60 | -2.39 | 8.49E-17 | 4.72E-16 | 1.69E-11 |
| HALLMARK_INTERFERON_ALPHA_RESPONSE | -0.70 | -2.52 | 4.24E-15 | 2.12E-14 | 7.59E-10 |
| HALLMARK_MYC_TARGETS_V2 | 0.77 | 2.86 | 7.90E-15 | 3.59E-14 | 1.28E-09 |
| HALLMARK_COAGULATION | -0.64 | -2.41 | 9.66E-15 | 4.03E-14 | 1.44E-09 |
| HALLMARK_MYOGENESIS | -0.56 | -2.21 | 1.74E-12 | 6.69E-12 | 2.40E-07 |
| HALLMARK_IL2_STAT5_SIGNALING | -0.55 | -2.16 | 4.72E-12 | 1.69E-11 | 6.04E-07 |
| HALLMARK_APICAL_JUNCTION | -0.54 | -2.13 | 5.29E-11 | 1.76E-10 | 6.31E-06 |
| HALLMARK_KRAS_SIGNALING_UP | -0.54 | -2.13 | 9.55E-11 | 2.99E-10 | 1.07E-05 |
| HALLMARK_IL6_JAK_STAT3_SIGNALING | -0.64 | -2.29 | 4.82E-10 | 1.42E-09 | 5.07E-05 |
| HALLMARK_HYPOXIA | -0.52 | -2.06 | 1.19E-09 | 3.30E-09 | 1.18E-04 |
| HALLMARK_MTORC1_SIGNALING | 0.45 | 2.05 | 6.49E-09 | 1.71E-08 | 6.11E-04 |
| HALLMARK_XENOBIOTIC_METABOLISM | -0.49 | -1.95 | 4.05E-08 | 1.01E-07 | 3.63E-03 |
| HALLMARK_UV_RESPONSE_DN | -0.54 | -2.02 | 5.54E-08 | 1.32E-07 | 4.72E-03 |
| HALLMARK_MITOTIC_SPINDLE | 0.43 | 1.95 | 8.19E-08 | 1.86E-07 | 6.66E-03 |
| HALLMARK_APICAL_SURFACE | -0.70 | -2.20 | 2.10E-07 | 4.56E-07 | 1.63E-02 |
| HALLMARK_APOPTOSIS | -0.50 | -1.93 | 2.52E-07 | 5.26E-07 | 1.88E-02 |
| HALLMARK_OXIDATIVE_PHOSPHORYLATION | 0.42 | 1.91 | 4.15E-07 | 8.30E-07 | 2.97E-02 |
| HALLMARK_ANGIOGENESIS | -0.72 | -2.18 | 6.10E-07 | 1.17E-06 | 4.20E-03 |
| HALLMARK_DNA_REPAIR | 0.44 | 1.91 | 2.10E-06 | 3.89E-06 | 1.39E-01 |
| HALLMARK_ESTROGEN_RESPONSE_EARLY | -0.41 | -1.62 | 2.84E-04 | 5.08E-04 | 1.82E-04 |
| HALLMARK_TGF_BETA_SIGNALING | -0.54 | -1.77 | 9.72E-04 | 1.68E-03 | 6.00E-04 |
| HALLMARK_UNFOLDED_PROTEIN_RESPONSE | 0.38 | 1.57 | 2.10E-03 | 3.49E-03 | 1.25E-03 |
| HALLMARK_NOTCH_SIGNALING | -0.56 | -1.66 | 6.44E-03 | 1.04E-02 | 3.72E-03 |
| HALLMARK_P53_PATHWAY | -0.34 | -1.36 | 1.83E-02 | 2.85E-02 | 1.02E-02 |

NES: Normalized Enrichment Score; p.adjust: Adjusted p-value
